# Prescription of antibiotics for urinary tract infections in outpatient care in Bavaria: An analysis of routine data

**DOI:** 10.1371/journal.pone.0312620

**Published:** 2024-10-25

**Authors:** Thomas Hanslmeier, Sahera Alsaiad, Susann Hueber, Peter K. Kurotschka, Roman Gerlach, Ildikó Gágyor, Yvonne Kaußner

**Affiliations:** 1 Department of General Practice, University Hospital Würzburg, Würzburg, Bavaria, Germany; 2 Institute of General Practice, University Hospital Erlangen, Erlangen, Bavaria, Germany; 3 Kassenärztliche Vereinigung Bayerns (KVB), Bavaria, Germany; 4 Counseling Center for Employees, University Hospital Wuerzburg, Bavaria, Germany; Kirklareli Universitesi, TÜRKIYE

## Abstract

**Background:**

Patients with urinary tract infection (UTI) in German outpatient care are usually treated by general practitioners (GPs), as well as by other specialties. To prevent antibiotic resistances and side effects, German guidelines recommend fosfomycin, nitrofurantoin, pivmecillinam and nitroxoline as first-line treatments, and advice against broad-spectrum antibiotics such as fluoroquinolones and cephalosporins. However, data from the European Centre for Disease Prevention and Control indicates a significant proportion of second-line antibiotics in German outpatient care. Our aim was to analyze whether antibiotic prescription has changed over time in accordance with guidelines. In addition, we aimed to investigate whether specialties prescribe different antibiotics for UTIs and whether prescription varies according to patient age and sex. For patients receiving more than one antibiotic, we wanted to determine whether subsequent prescriptions show a change in substances and specialties involved.

**Methods:**

This retrospective study involved routine data (2013 to 2019) provided by the Bavarian Association of Statutory Health Insurance Physicians. Data on diagnoses and prescriptions were transmitted from outpatient care physicians on a quarterly basis. UTI patients ≥12 years were included.

**Results:**

We analyzed 1.7 million UTI prescription cases. In females, shares of fluoroquinolones decreased sharply over time, while shares of first-line substances fosfomycin and pivmecillinam increased. Gynecologists showed the highest shares of first-line substances compared to GPs and urologists. Fluoroquinolone shares decreased in all three specialty groups. In females, older patients showed lower shares of first-line substances than younger patients. If a second or third antibiotic was prescribed, fosfomycin shares decreased, while shares of nitrofurantoin, nitroxoline and cephalosporins increased.

**Conclusions:**

Our findings show a trend towards a more guideline-adherent prescribing in the treatment of UTI, with a significant increase of shares of fosfomycin and pivmecillinam, especially in women, and a sharp decrease of shares of fluoroquinolones.

## Introduction

Urinary tract infection (UTI) is a common condition, especially in women who have an annual prevalence of 11% and a lifetime prevalence of at least 50% [1, 2]. In German outpatient care, UTI are treated not only by general practitioners (GPs) but also by other resident specialists, i.e., gynecologists and urologists. Patients in German outpatient care have free choice of doctor and can consult as many GPs and subspecialists as they want [3].

Antibiotics are prescribed in 71–95% of female UTI cases [2–5]. Most European guidelines recommend the use of antibiotics in treatment of UTIs [4]. UTIs subside earlier under antibiotic therapy compared to alternative approaches, with a lower overall symptom burden [5–9]. The German interdisciplinary S3-guideline on uncomplicated UTI recommends the prescription of fosfomycin, pivmecillinam, nitrofurantoin and nitroxoline for first-line treatment of female patients. In addition, the prescription of trimethoprim is recommended in regions with resistance rates < 20% [10, 11]. While fluoroquinolones were widely used for UTI treatment in the past [12], new data on possible severe side effects caused by fluoroquinolones (among others: aortic dissection, aortic aneurysm, tendinitis) led to two dear-doctor letters in 2018 and 2019 in Germany calling for restraint in the use of these substances [13, 14]. Dear-doctor letters (‘Rote-Hand-Briefe’) are sent out by pharmaceutical companies informing healthcare professionals of newly identified drug-associated risks. As a result of these new findings, fluoroquinolones are no longer recommended as first-line treatment for uncomplicated UTIs in females. Meanwhile, with pivmecillinam a new substance for UTI treatment was introduced to the German market in 2016 [10].

For male patients the German guidelines recommend the use of pivmecillinam and nitrofurantoin for treatment of UTI [10]. For pregnant patients with uncomplicated UTI, the guidelines recommend the use of penicillin, cephalosporins or fosfomycin [10, 11]. For adolescents ≥12 years with uncomplicated cystitis the German pediatric guidelines recommend the use of fosfomycin, nitroxoline, trimethoprim and amoxicillin / clavulanic acid [15].

In order to prevent the emergence of new antibiotic resistances and the occurrence of unnecessary side effects, it is of great importance that only substances suitable for the treatment of UTI are prescribed. Data of the European Centre for Disease Prevention and Control indicates a high share of broad spectrum antibiotics like fluoroquinolones in German outpatient care for the years of 2013 to 2022 compared to other European countries [16].

Considering the overall high consumption of antibiotics caused by UTI and the extensive changes in recommendations within the last years, the present study aimed to (1) provide an overview on how frequently UTI diagnoses have been coded by which specialists in outpatient care in Bavaria and to analyze (2) which antibiotics have been prescribed in UTI treatment. The aim was to find out whether the prescription patterns show changes over time in accordance with the revised recommendations of German interdisciplinary S3-guidelines. Furthermore, we wanted to analyze (3) if physicians of different specialties treat UTIs differently and (4) if prescription patterns vary depending on patient age and sex. (5) In patients who received more than one antibiotic to treat the same UTI we aimed to determine, whether subsequent prescriptions differed in the choice of substances and in the specialty groups involved.

## Materials & methods

### Ethics

For this retrospective analysis of routine data, we have received a waiver by the Ethics Committee of the University of Wuerzburg on December 18, 2019 (20191021 02). Data were anonymized (see below). We did not have access to information that could identify individual patients at any time. Data was accessed for research purposes between November 6, 2020 and June 14, 2024.

### Data source

Routine data were provided by the Bavarian Association of Statutory Health Insurance Physicians (Kassenärztliche Vereinigung Bayerns, KVB). Data on diagnoses and prescriptions were transmitted from outpatient care physicians to the KVB on a quarterly basis. The dataset contains UTI diagnoses and antibacterial prescriptions for patients in Bavarian outpatient care over a period of seven years (2013–2019) with information on 2.2 million Bavarian patients who had least one confirmed UTI diagnosis following the International Classification of Diseases (10th revision; ICD-10: N39.0: urinary tract infection, site not specified, N30.0: acute cystitis) within the study period. With 13.1 million inhabitants (2019), Bavaria is the federal state with the second most inhabitants in Germany, accounting for 15.8% of the German population (83.2 million in 2019) [17].

Next to confirmed diagnoses, the dataset also contains information on infections that were not confirmed, but only suspected and infections that already lay in the past (‘Status post’). Only confirmed diagnoses were included in this study. Data were anonymized, not including patients’ names and addresses. Instead, a numerical patient id was assigned to every patient, allowing their assignment to the UTI cases throughout the dataset. Data includes demographic information (patients’ sex, year and month of birth). Only patients with valid sex and age information aged ≥ 12 years were included.

### Frequencies of UTI diagnoses

The dataset does not show the exact date on which a diagnosis was coded by physicians but only the respective quarter. Thus, multiple diagnosis of patients could only be analyzed in aggregated form for a whole quarter. A UTI case was therefore defined as a patient with at least one UTI in one quarter. It was analyzed, how often the different UTI diagnoses (N39.0, N30.0) were coded by which specialties for female and male patients.

### Antibacterial treatment of UTI

For the analysis of prescription patterns, patients with at least one diagnosis of N39.0 or N30.0 were included. Prescription codes follow the anatomical therapeutic chemical classification (ATC) [18]. All antibacterials for systemic use (ATC-Codes starting with ‘J01’) were included. Only UTI cases with at least one antibiotic prescription in the same quarter of the UTI were included. UTI cases were excluded if prescription and diagnosis were not encoded by the same practice. The data do not contain information on the medical indication of prescriptions. If a patient case shows UTI and another infection outside the urinary tract (e.g. pneumonia) at the same time, it is not possible to determine, whether an antibiotic was prescribed for the treatment of the UTI or the other infection. To avoid wrong assignments of antibiotics to UTI treatment, cases with UTI (N39.0, N30.0) and another infection in the same quarter were excluded.

To determine how prescription patterns in UTI treatment changed over time, all prescriptions of UTI cases of the same year were summarized. Substances were then grouped as follows: cephalosporins (J01DB, J01DC, J01DD, J01DE), fluoroquinolones (J01MA), fosfomycin (J01XX01), nitrofurantoin (J01XE01), nitroxoline (J01XX07), pivmecillinam (J01CA08), trimethoprim (J01EA01), the combination of sulfamethoxazole and trimethoprim (J01EE01), and other antibacterials (all remaining ATC codes starting with J01). For further analysis, UTI cases were divided into subgroups according to physician’s specialty (GPs, urologists, gynecologists, others) and patient’s age (12–18, 20–29, 30–39, 40–49, 50–59, 60–69, 70–79, 80–89, >90 years) and sex (male, female).

### Prescriptions in UTI cases in chronological order

Some patients receive more than one antibiotic prescription for the treatment of the same UTI. We aimed to analyze whether the choice of antibiotic substances changed when a second or third antibiotic was prescribed. To ensure that the first prescription of a given UTI case was identified correctly, only those UTI cases in which no UTI was diagnosed in the four previous quarters were included for this analysis. Antibiotic prescriptions coded in the same quarter as the UTI and in the following quarter were analyzed. The dataset contains the day and month in which a prescription was coded. The prescriptions within each UTI case were sorted in chronological order. The prescriptions of all UTI cases were then merged into three groups as follows: The first prescription of every UTI case was added to the group ‘first prescription’. If a UTI case contained only one prescription, it was added to the group ‘first prescription’. If a case showed more than one prescription, the second prescription was added to the group ‘second prescription’. If a case showed more than two prescriptions, the third prescription was added to the group ‘third prescription’. Two prescriptions on the same day were assigned to the same group. Prescriptions were excluded if they followed the previous prescription in more than 14 days, as these prescriptions were likely intended for the treatment of another, newly emerging infection. For the resulting three groups (first, second, third prescription), it was analyzed which antibiotic substances were prescribed and which specialties were involved. In rare cases, UTI cases showed more than three prescriptions. Fourth and more prescriptions were not analyzed due to very small overall numbers (n = 344 prescriptions).

## Results

### UTI cases

In total, 2.2 million patients were diagnosed with at least one UTI (ICD-Codes: N39.0, N30.0) between 2013 and 2019, which led to a total of 5.9 million UTI-diagnoses. Most UTIs were encoded in female patients (N39.0: 82.1%, N30.0: 80.7% female patients). GPs, gynecologists and urologists together accounted for 92% of all N39.0-diagnoses in females. The ICD-Code N39.0 was diagnosed by GPs in 72%, by urologists in 12% and by gynecologists in 8% of all cases (Table 1).

**Table 1:** UTI diagnoses by sex and physician specialty (2013–2019).

	Total	physician specialty				
		GPs*	Urologists	Gynecologists	Internal medicine physicians	Other specialties
Female patients						
Urinary tract infection, site not specified (N39.0)	4.857.944	2.960.629	510.334	339.285	127.424	193.592
	(100.0%)	(71.7%)	(12.4%)	(8.2%)	(3.1%)	(4.7%)
Acute cystitis (N30.0)	726.680	361.918	200.282	116.567	13.575	34.338
	(100.0%)	(49.8%)	(27.6%)	(16.0%)	(1.9%)	(4.7%)
Male patients						
Urinary tract infection, site not specified (N39.0)	897.679	508.999	315.768	322	25.571	47.019
	(100.0%)	(56.7%)	(35.2%)	(0.0%)	(2.8%)	(5.2%)
Acute cystitis (N30.0)	173.980	49.057	116.546	144	2.816	5.417
	(100.0%)	(28.2%)	(67.0%)	(0.1%)	(1.6%)	(3.1%)

*Diagnoses encoded by internal medicine specialists working as GPs (‘hausärztliche Internisten’) were added to the group of GPs not to the group of internal medicine specialists.

### Changes in antibacterial prescription between 2013 and 2019

**Fig 1:**
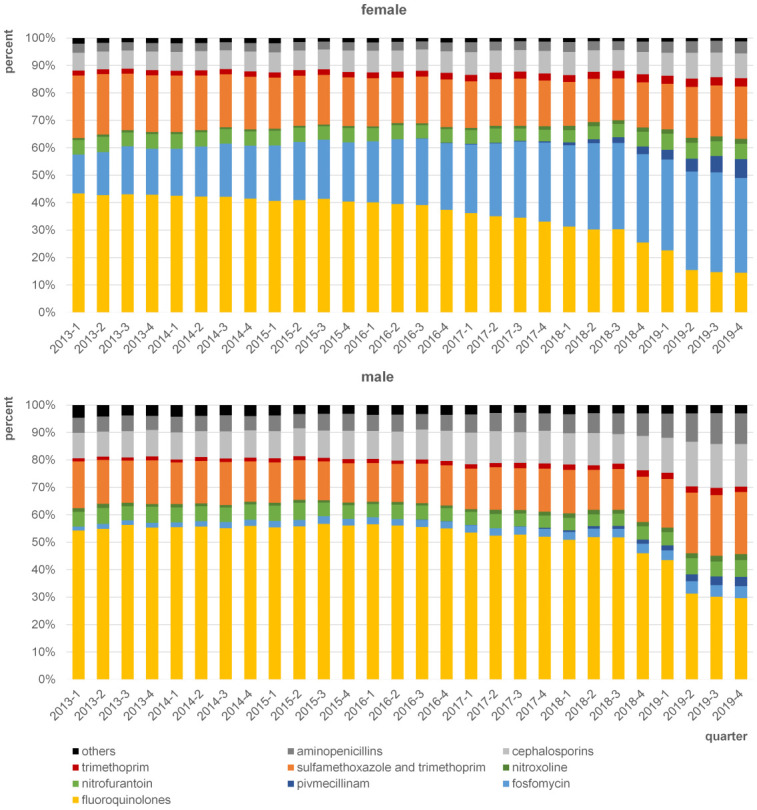
Shares of antibiotic substances on total prescription by quarter from 2013 to 2019 for female and male patients with UTI (N39.0, N30.0)(female: n = 1.681.255 prescriptions; male: n = 269.151prescriptions).

The analysis included 1.7 million UTI prescription cases. The number of prescriptions for the treatment of UTI remained relatively constant within the sample, with seasonal fluctuations (see S1 Fig). Fig 1 shows the shares of different substance groups on total antibiotic prescription for UTI treatment in each quarter. Prescriptions of all physician specialties in outpatient care are included. In female patients, shares of fosfomycin prescription increased from 14.1% in the first quarter of 2013 to 34.5% in the last quarter of 2019. Shares of pivmecillinam also increased within the study period, from 0.0% in the first quarter of 2013 to 6.9% in the last quarter of 2019, whereas the shares of fluoroquinolones decreased from 43.4% in the first quarter of 2013 to 14.4% in the last quarter of 2019. A decrease in the shares of fluoroquinolones was also observed among male patients, from 54.3% in the first quarter of 2013 to 29.6% in the last quarter of 2019. Compared to female patients, fosfomycin shares remained on a relatively low level in male patients, between 1.4% to 4.5%.

### Development of antibacterial treatment by physician specialty

**Fig 2:**
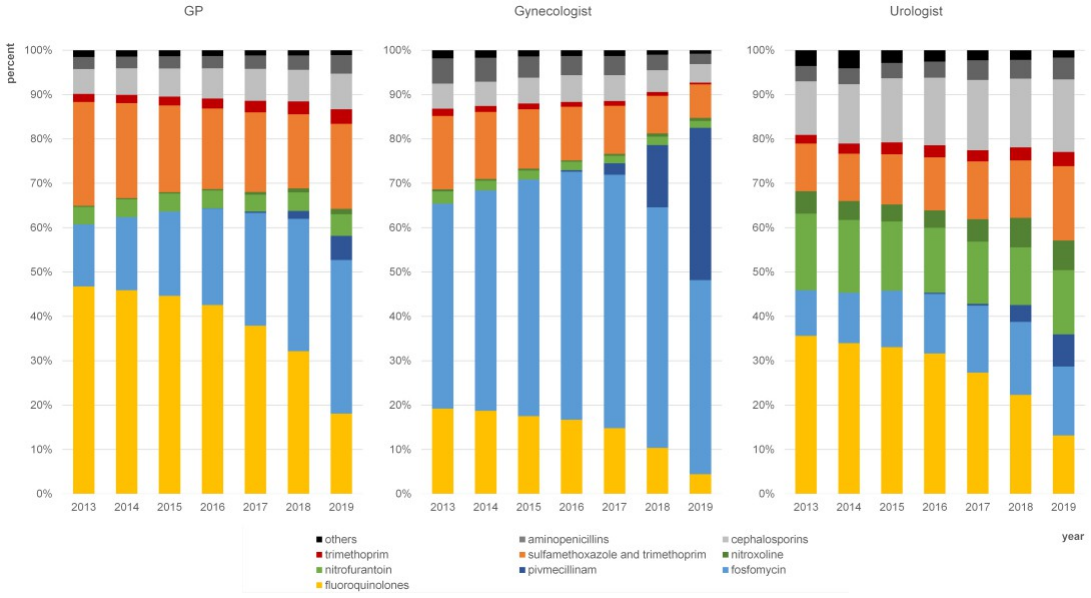
Shares of antibiotic substances on total prescription in female patients with UTI (N39.0, N30.0) by physician specialties (GPs: n = 1.299.641 prescriptions; gynecologists n = 141.814 prescriptions; urologists n = 182.100 prescriptions).

Fig 2 shows prescription patterns of the three specialties who diagnosed UTI most often: GPs, gynecologists, and urologists. For all three physician specialties a decrease of shares of fluoroquinolones could be observed within the study period, while shares of the first-line substances fosfomycin and pivmecillinam increased in all three specialties over time. Gynecologists showed the overall highest shares of fosfomycin and pivmecillinam and the lowest shares of fluoroquinolones compared to GPs and urologists. Within the group of first-line substances, urologists tended to prescribe nitrofurantoin and nitroxoline more often than GPs and gynecologists. The shares of the combination of sulfamethoxazole and trimethoprim decreased notably only in gynecologists. Urologists showed comparatively high shares of cephalosporins.

### Antibacterial treatment by age and sex

**Fig 3:**
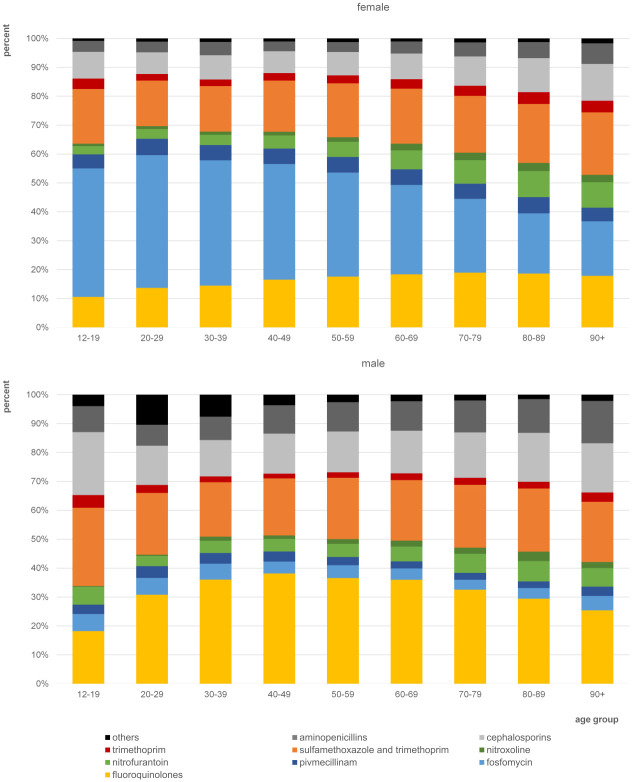
Shares of antibiotic substances on total prescription in UTI treatment (N39.0, N30.0) by sex and age group in 2019 (female: n = 238.670 prescriptions; male: n = 40.641 prescriptions).

The comparison of prescription shares by age group showed a continuous decline in the shares of fosfomycin among women with increasing age, starting from age group 20–29 years (45.9% in age group 20–29 years to 18.9% in age group 90+ years) (Fig 3). Fluoroquinolone shares increased in females with increasing age from 10.5% in age group 12–19 years to 19.0% in age group 70–79 years. In males, fluoroquinolone shares were highest in the age group of 40–49 years (38.1%) and then decreased continuously with increasing age (25.4% in age group 90+ years). The age group of 12–19-year-old patients shows the lowest share of fluoroquinolones in males, while shares of cephalosporins and the combination of sulfamethoxazole and trimethoprim were comparably high.

### Prescriptions in UTI cases in chronological order

**Fig 4:**
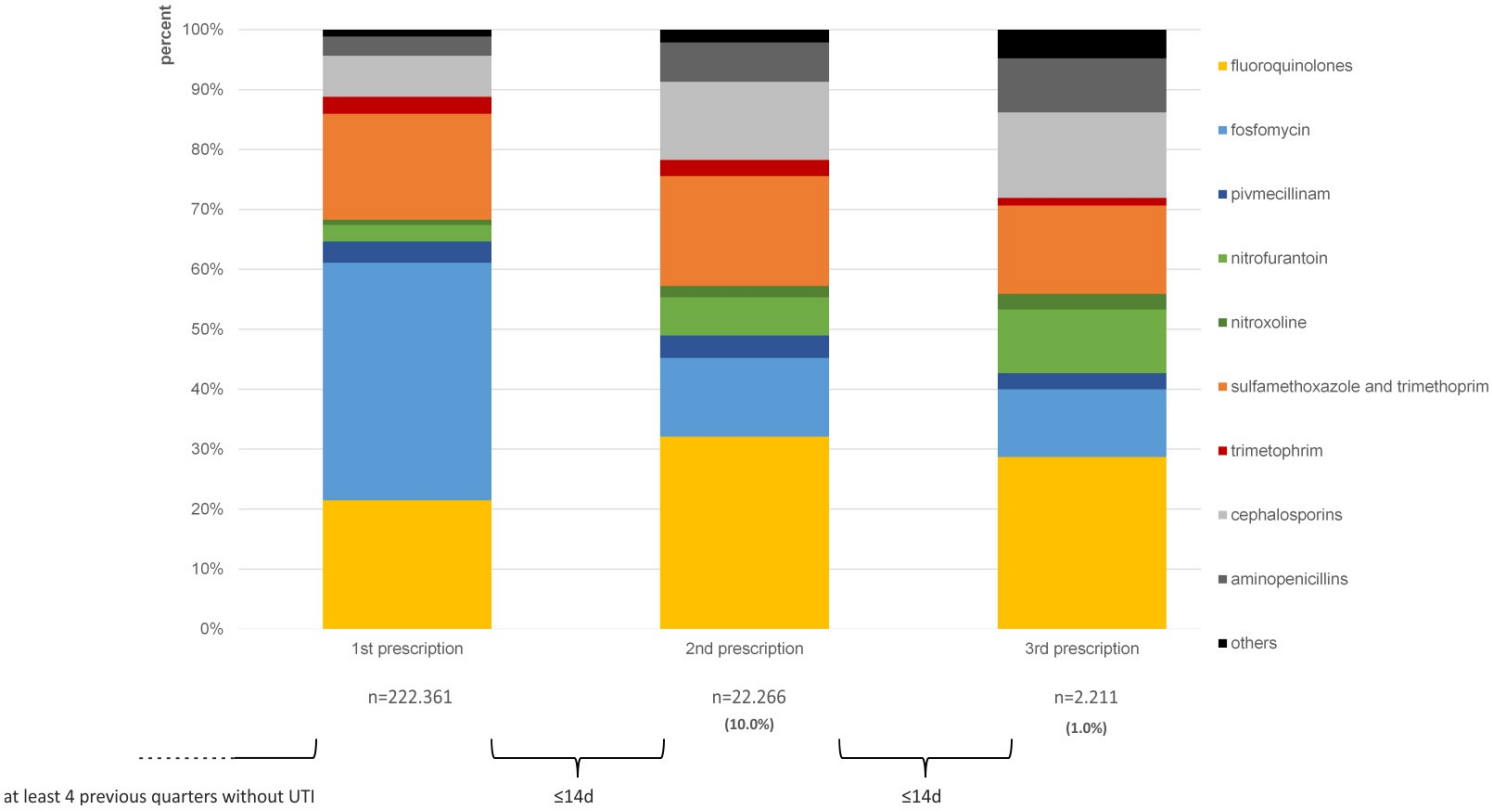
Comparison of 1^st^, 2^nd^, and 3^rd^ antibiotic prescription. Shares of antibiotic substances on total prescription in female UTI cases (N39.0, N30.0) in 2018 and 2019.

Some patients receive more than one antibiotic prescription for the treatment of the same UTI. We wanted to analyze whether the antibiotic substances changed when a second or third antibiotic was prescribed. Fig 4 shows antibiotic prescriptions of UTI cases in chronological order for female UTI patients. Column one shows the first antibiotic that was prescribed within UTI cases. The analysis shows a total of 222.361 first prescriptions. 22.266 second prescriptions (10.0% of cases) within the following 14 days were recorded. In 1.0% of cases, a third prescription was recorded within 14 days (2.211 prescriptions). The columns show the shares of different substance groups on total antibiotic prescription. Second and third prescriptions show lower shares of fosfomycin and pivmecillinam compared to the first prescription column, while shares of nitroxoline, nitrofurantoin, cephalosporins and aminopenicillins were comparably higher.

Regardless of whether it was the first, second or third prescription, most antibiotics were prescribed by GPs. The share of antibiotics prescribed by GPs remained on a relatively high level with 81% in first prescriptions to 75% in third prescriptions (see S2 Fig). The shares of gynecologists were higher in the third prescription group than in first prescription group. Shares of urologists were higher in the first prescription group than in the groups of second and third prescription.

## Discussion

Antibiotic prescriptions of 1.7 million outpatient UTI cases in the federal state of Bavaria were analyzed based on routine data. While the overall number of prescriptions for UTI treatment remained on a constant level over the years, the different types of antibiotics were subject to substantial changes: while fluoroquinolones made up the largest share among all substance groups in 2013, their shares fell sharply in the following years in both men and women. A significant increase for shares of first-line substances fosfomycin and pivmecillinam was observed, particularly for female patients. In this regard, our findings indicate a trend towards a more guideline-adherent prescription. The specialty groups differ in their choice of substances. Gynecologists showed the highest shares of first-line substances. Within the group of first-line substances, urologists prescribed nitrofurantoin and nitroxoline more frequently than GPs and gynecologists. Fluoroquinolone shares decreased within all three specialty groups. In females, older UTI patients were less often prescribed first-line substances than younger patients. Male UTI patients showed higher shares of cephalosporins, and fluoroquinolones compared to female patients in their respective age group. If a second or third prescription was necessary for UTI treatment, fosfomycin was prescribed less often compared to the first prescription, while shares of nitrofurantoin, nitroxoline and cephalosporins increased.

Two studies have investigated the treatment of UTI in Germany in recent years using routine data: Dicheva (2015) has analyzed nation-wide insurance data of female UTI patients for the years 2012 and 2013 [12]. Schmiemann et al. (2022) have analyzed insurance data for the federal state of Bremen for female and male patients from 2015–2019 [19]. Regarding the development of prescription patterns over time our data show a steady decrease of fluoroquinolone shares between 2013 and 2019 in both females and males. While fluoroquinolones were the substance group most often prescribed in outpatient care in 2013 (43%) in females, its shares decreased to 14% in 2019. Other studies confirm this trend: According to Dicheva, fluoroquinolones were the substance group that accounted for the largest share on total prescriptions in UTI treatment in 2013 [12]. The decrease in fluoroquinolone prescriptions seen in the present work was also shown by Schmiemann et al. for the federal state of Bremen [19]. As in Bavaria, shares of fluoroquinolones in Bremen decreased most sharply in the years of 2018 and 2019. The decrease of fluoroquinolone prescriptions in UTI treatment seems to be part of a larger development, since the overall prescription of fluoroquinolones decreased in all Germany during these years [20, 21]. This might have been due to an increased awareness among physicians for possible severe side effects of fluoroquinolones. Two dear-doctor letters have informed German physicians about side effects of fluoroquinolones in 2017 and 2018 [13, 14]. The publication of an updated guideline for the treatment of uncomplicated UTI in Germany in April 2017, where fluoroquinolones are not recommended for first-line treatment of uncomplicated UTI in women [10], might also have contributed to the decrease of fluoroquinolone shares in UTI treatment. While fluoroquinolone shares decreased over time, fosfomycin shares increased especially for female patients. Pivmecillinam shares increased in both men and women since 2016, the year it has been introduced in Germany. Findings from Schmiemann et al. also showed an increase for these two substances for the federal state of Bremen [19]. The rise of shares of the two substances fosfomycin and pivmecillinam, both of which are recommended as first-line treatment for uncomplicated UTI in women, and the decline of fluoroquinolone shares suggest a trend towards a more guideline adherent prescription among Bavarian physicians. However, shares of the combination of sulfamethoxazole and trimethoprim remained on a constant high level in females, even though it is not recommended anymore for the treatment of uncomplicated UTI in females by the German interdisciplinary S3-guidelines [10].

Prescription patterns of different physician specialties significantly differ in the treatment of UTI in females. Regarding the first-line substances, gynecologists most often used fosfomycin and also showed comparably high shares of pivmecillinam in the most recent years, while urologists prescribed nitrofurantoin and nitroxoline much more often than the other two specialties. The different specialist groups appear to have different preferences within the group of first-line substances. With pivmecillinam, shares have increased in all three specialties since its introduction on the market, with the highest shares seen in gynecologists. The substance seems to increasingly establish in UTI treatment. All three specialties showed a decrease in fluoroquinolone shares. Shares of sulfamethoxazole and trimethoprim, not recommended as first-line substance by German interdisciplinary S3-guidelines, show a notable decrease only in gynecologists.

Regarding UTI prescriptions by age group, our findings show lower shares of fosfomycin for older patients compared to younger adult patients, while shares of nitrofurantoin and nitroxoline are comparably higher in older female patient groups. Also, shares of fluoroquinolones are comparatively higher in older female UTI patients. Findings of Schmiemann et al. (2023) show the same trends for female adult patients in Bremen. These differences between age groups might be due to the fact that older patients suffer more frequently from recurring or relapsing UTIs. In older patients commonly used first-line substances like fosfomycin or pivmecillinam may have already been tried before without success, leading physicians to prescribe less commonly used first-line substances like nitroxoline and nitrofurantoin or second-line substances like fluoroquinolones.

Some patients received more than one prescription for the treatment of the same UTI. In the groups of second and third prescriptions, fosfomycin shares were lower compared to the group of first prescriptions, while other substances like nitrofurantoin, fluoroquinolones, and cephalosporins were comparably higher. As above, commonly used first-line substances like fosfomycin might have already been tried without success. Therefore, less commonly used first-line substances like nitroxoline and nitrofurantoin or second-line substances like fluoroquinolones and cephalosporins may have been prescribed.

### Strengths and limitations

The use of routine data enabled the analysis of a very large patient group (1.7 million UTI prescription cases) within Bavarian outpatient care over a long period of time (2013 to 2019). However, routine data are primarily collected for billing purposes, not for research, which raises the question of data accuracy and completeness. To improve data accuracy, we excluded data of patients with inconsistent information on age and sex. German routine data offer no direct link between prescriptions and diagnoses. Antibiotic prescriptions that are coded in the same quarter as a UTI might also have been prescribed for the treatment of another infection outside the urinary tract. To exclude antibiotics that were prescribed for the treatment of another infection, several filters were implemented as follows. Only prescriptions in the same quarter coded by the same practice that had also coded the UTI diagnosis were included. UTI patients with other infections in the same quarter were excluded.

## Conclusions

Within 2013 and 2019 shares of the first-line substances fosfomycin and pivmecillinam showed a significant increase in the treatment of UTI especially in females, while shares of fluoroquinolones decreased sharply. However, shares of the combination of sulfamethoxazole and trimethoprim remained on a constant high level in females, even though it is not recommended anymore for first-line treatment of uncomplicated UTI by the German guidelines. While our findings on prescription patterns in UTI treatment suggest a clear trend towards a greater guideline adherence in recent years, further antibiotic stewardship interventions should be undertaken to further improve guideline adherence.

## Supporting information

S1 Fig Number of antibiotic prescriptions in female and male patients with UTI (N30.0 and/or N39.0). (TIF)

S2 Fig Shares of specialist groups prescribing 1^st^, 2^nd^, and 3^rd^ antibiotic prescriptions in UTI treatment of females (N39.0, N30.0) in 2018 and 2019. (TIF)
