# Supplementary figures and images for: Prescription of antibiotics for urinary tract infections in outpatient care in Bavaria: An analysis of routine data
Source: PLoS One. 2024 Oct 25;19(10):e0312620. doi: 10.1371/journal.pone.0312620 (PMC11508671; doi:10.1371/journal.pone.0312620)

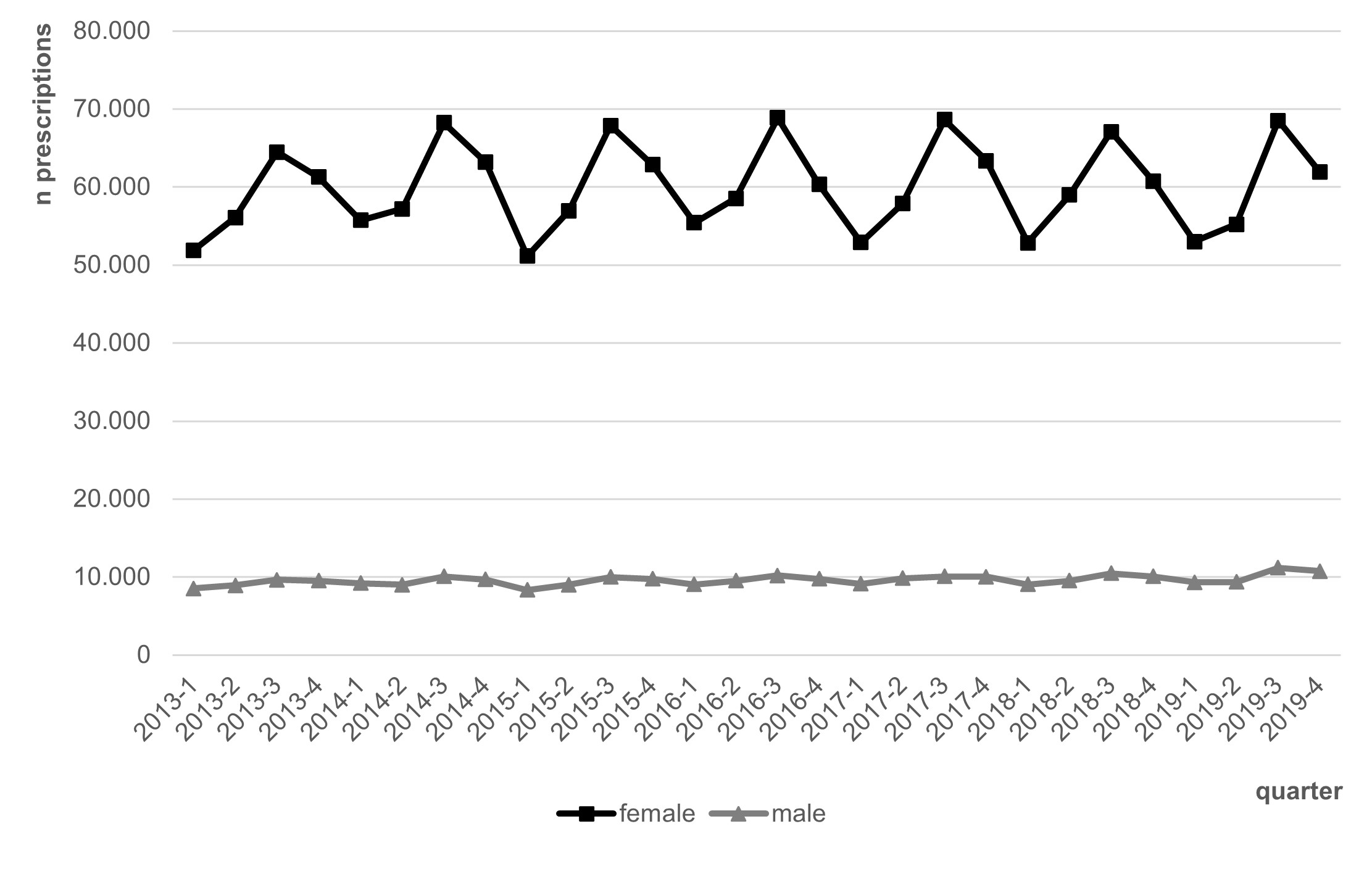

Supplement: S1 Fig — (TIF) [file pone.0312620.s001.tif]

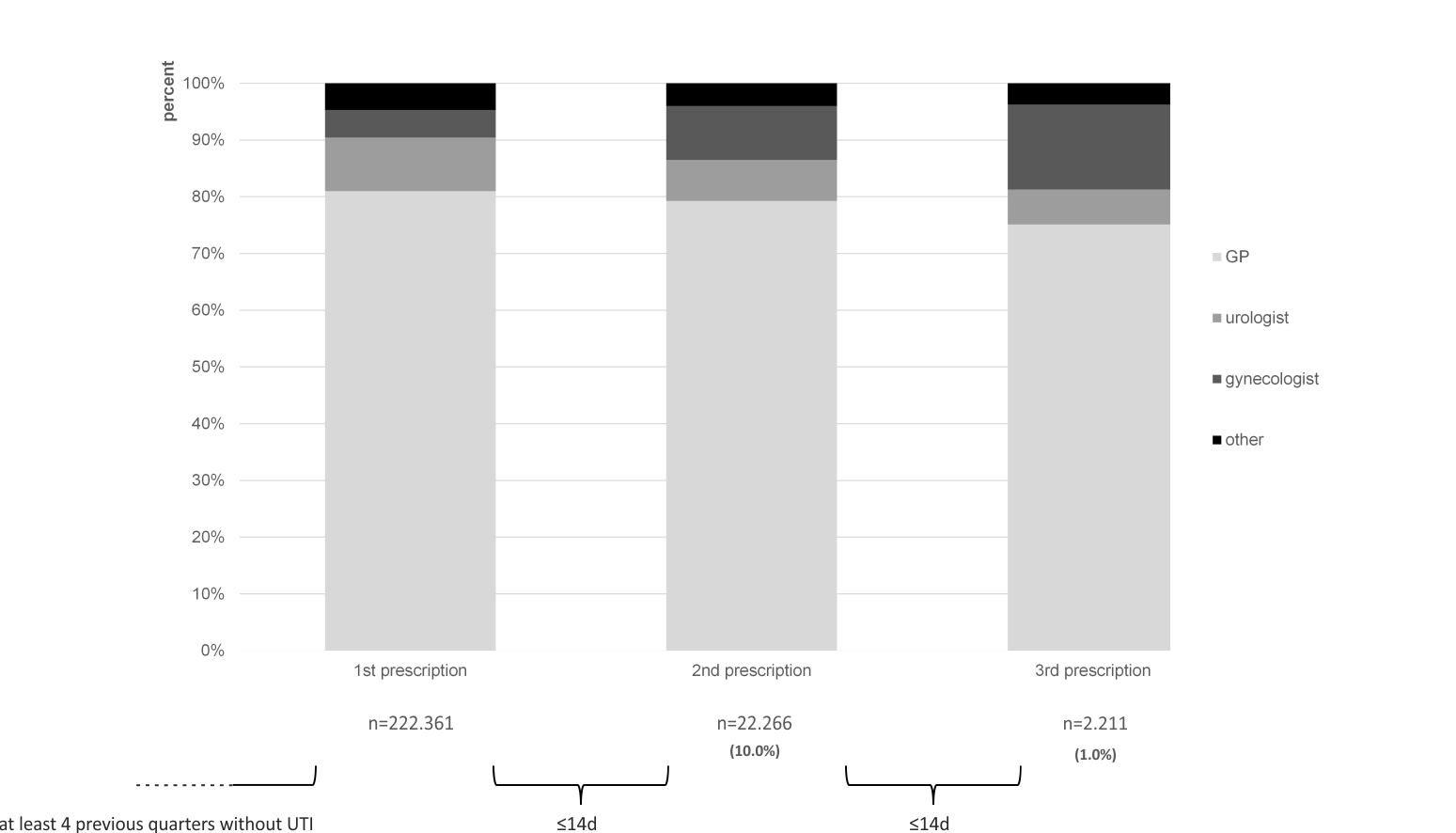

Supplement: S2 Fig — (TIF) [file pone.0312620.s002.tif]
